# Home-visiting programs based on the Brazelton approach: a scoping review

**DOI:** 10.1007/s00431-023-05048-3

**Published:** 2023-06-07

**Authors:** Cecilia Tazza, Salvatore Ioverno, Susanna Pallini

**Affiliations:** Department of Education, Roma Tre University, Via del Castro Pretorio 20, Rome, 00185 Italy

**Keywords:** Home visiting, Neonatal behavioral observation, Anticipatory guidance, Brazelton, Scoping review

## Abstract

This review maps and summarizes the quantitative studies on the main outcomes associated with home visiting (HV) programs using Brazelton methods aimed at supporting expectant and new parents. One hundred thirty-seven records were identified, and 19 records were selected. The design of our study was based on the methodological framework for conducting scoping reviews. Quality was assessed through the Jadad scale. Studies were coded for participant characteristics (number, mean age, and risk status), methodology (recruitment, home visit frequency, age of the child, Brazelton method, and research design), and intervention outcomes (on infants, parents, and home visitors). The studies mostly focused on the impact of Brazelton HV programs on infant development, the mother’s psychological well-being, mother-infant interaction, and home visitor satisfaction. Experimental and quasi-experimental studies consistently have shown that parents receiving the intervention have greater knowledge of their children. Results are less conclusive regarding the intervention’s impact on other domains of child development, mothers’ psychological well-being, and mothers’ sensitivity to the relationship with the child. Overall, the results suggest that the improvements associated with the intervention are mainly influenced by the risk status of families. Further research is needed to better understand the benefits of HV based on the Brazelton approach and the target population that may benefit most from this intervention.

*Conclusion*: Although the impact of the Brazelton home visiting intervention is not yet fully understood, there is promising evidence of its positive effects on child development, maternal well-being, and parental knowledge. Further research with consistent methodologies and larger sample sizes is needed to strengthen our understanding. However, existing findings in the literature underscore the importance of preventive interventions such as the Brazelton program in improving family well-being, with potential long-term benefits.

**Table:** 

**What is Known:**
• *Home visiting programs based on the Brazelton approach aim to increase parents’ knowledge and sensitivity to their children.*
• *There is no clear picture in the literature of the effectiveness of these programs.*
**What is New:**
• *Existing studies consistently show the effectiveness of these programs to improve parents’ knowledge of their children.*
• *Findings on the impact of these programs on child development, mothers' psychological well-being and sensitivity to their child are inconclusive and may be influenced by the risk status.*

**Supplementary Information:**

The online version contains supplementary material available at 10.1007/s00431-023-05048-3.

## Introduction

The transition to parenthood and the beginning of a new life pose great challenges for parents and children, especially under risky conditions [1–3]. The scientific literature has identified home visiting programs as an effective strategy to guide parents to effectively address these challenges [4–9]. The term home visiting (HV) is typically used to refer to the various service programs provided by home carers or home visitors aimed at improving children’s development through parental support in their homes [10]. Although they are recommended treatments, their effectiveness depends on several factors, such as different implementation methods, the target of the intervention, the duration, the quality of training of the home visitors, the frequency of HVs, and the content of the curriculum of the intervention [1, 6].

A specific type of HV program is based on the Brazelton approach which aims to increase parental awareness and consequently sensitivity to child development and needs [11]. Brazelton identified specific, critical, and predictable developmental periods, called *touchpoints,* in which children learn new skills but at the same time exhibit increased nervousness and behavioral regressions that challenge parent–child interaction. Although these critical periods are functional in children’s growth [11], if not properly identified and anticipated, they can increase parents’ stress levels and decrease their sensitivity to children’s needs and parental self-efficacy [11, 12]. To support parents, Brazelton devised anticipatory guidance (AG) which leads parents to anticipate the various stages of their children’s development and related problem behaviors [11]. For example, the HV visitor can anticipate to parents their 9-month-old child’s new motor skills and the possibility that the child may be restless because he or she is channeling energy to begin walking [11, 13]. This methodology helps parents to understand their child’s development [13, 14], acquire new parenting skills [15], and avoid anxious overreactions [11, 13].

In the Brazelton approach, the home visitor may also use the Newborn Behavioral Assessment Scale (NBAS) or the Newborn Behavioral Observations system (NBO), which are protocols of systematical observation of infants’ responses to stimuli. They are administered in the presence of parents and guide them to find new ways of observing their children, focusing on their strengths, peculiarities, and temperament. These procedures are aimed at (a) creating a relationship between home visitors and parents that is collaborative, nonjudgmental, empathic, respectful, and aimed at identifying the family’s resources and strengths; (b) connecting parents with social resources; and (c) increasing parents’ understanding of their children and subsequent parental self-efficacy [16].

In 2018, Barlow and colleagues [17] reviewed 16 studies of Brazelton programs conducted in the USA. However, the authors did not distinguish which are provided at home and which are provided in a hospital or clinic, despite the different costs of the two types of interventions. Thus, to our knowledge, there are no reviews specifically examining the effectiveness of the Brazelton approach using HV programs. A scoping review methodology [18] on HV programs based on the Brazelton approach may be useful in filling this gap in the research literature, discussing its effectiveness, formulating methodological considerations, and identifying future directions. Specifically, we aimed to answer the following research questions:

1. Are the different home visiting programs based on the Brazelton approach effective in the promotion of child and parental adjustment outcomes (e.g., child development, mother’s psychological well-being, quality of mother-infant interaction)?
2. Do these outcomes differ according to specific categories of at-risk families?
3. What are the characteristics of existing research studies (e.g., participant demographics and methodological characteristics) on HV Brazelton programs?

## Materials and methods

### Study design

We conducted a scoping review, a type of review that aims to quickly map the key concepts underlying a research area and the main sources and types of evidence available [19]. The design of our study was based on Arskey and O'Malley’s [19] methodological framework for conducting scoping studies. Specifically, we proceeded through the following steps: identifying the research questions; identifying relevant studies; selecting studies; charting data; and collating, summarizing, and reporting results. In doing so, we followed the guidelines for PRISMA-ScR scoping reviews [20]. The collation, summary, and reporting of results aimed to use a descriptive approach to describe what improvements for children and/or parents are associated with Brazelton HV programs and whether these improvements can be categorized according to Brazelton technique, program beneficiary (e.g., parent or child), presence of a risk factor (e.g., maternal depression), and domain of adaptation (e.g., psychological well-being).

### Inclusion and exclusion criteria

Studies that reported all the four following characteristics were included in the review: (a) HV programs that used the Brazelton approach (i.e., touchpoint approach, NBO, anticipatory guidance, or NBAS) as the only approach or combined with other non-Brazelton approaches, (b) programs focused on supporting newborns or at-risk parents, (c) programs conducted by a practitioner who the authors explicitly stated was certified in the Brazelton approach, and (d) studies that evaluated the effectiveness of the program. Studies written in English, or another language understood by authors (French, Spanish, and Italian), and published in any date range were potentially eligible for inclusion.

Excluded were (a) studies in which Brazelton’s intervention was conducted in clinical or hospital settings (i.e., not in HV settings); (b) studies in which HV programs were not used as an intervention but as an evaluation of a treatment previously conducted in clinical or hospital settings; and (c) studies that used only a qualitative approach.

### Literature search

**Fig. 1:**
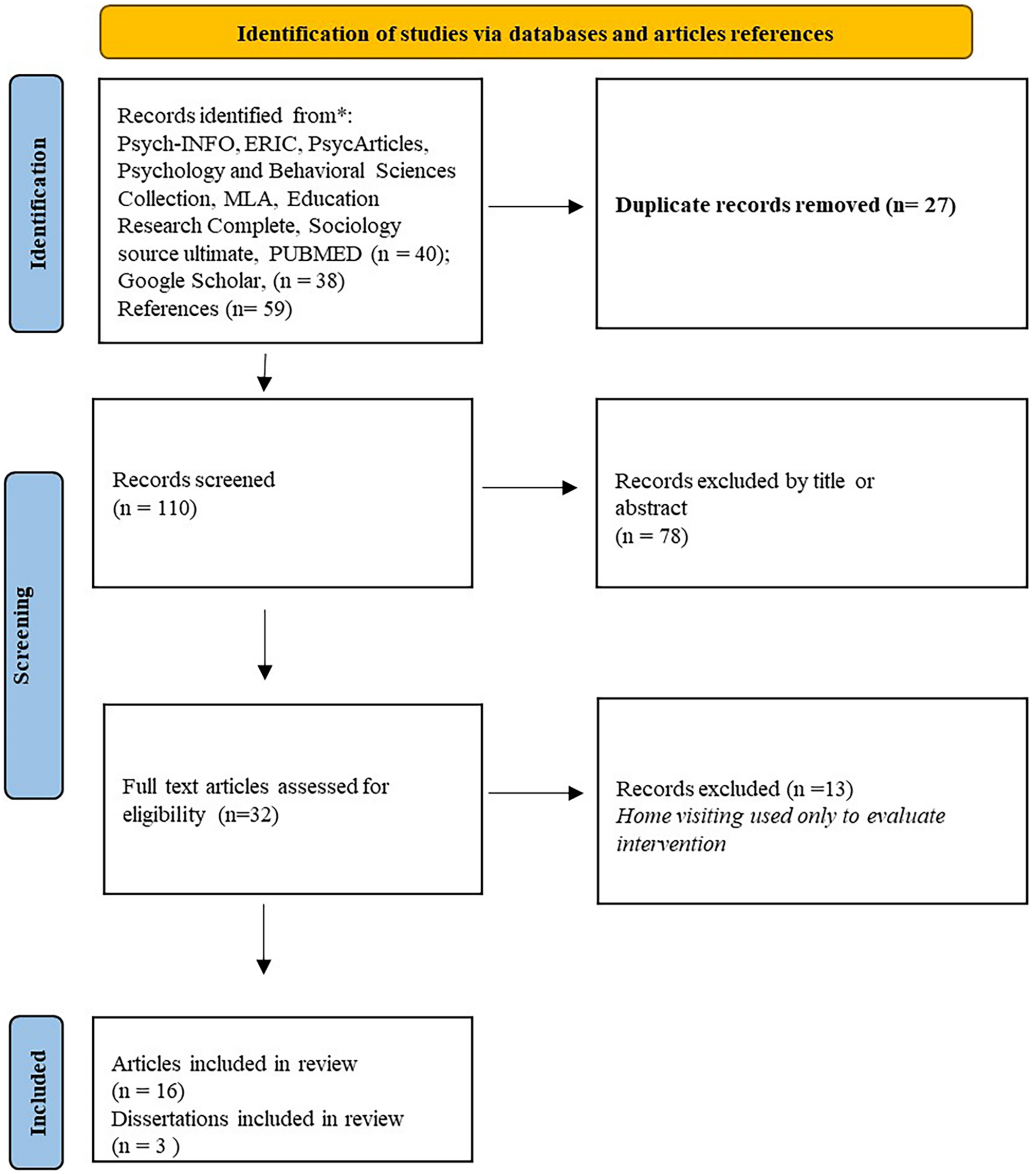
Identification of studies via databases and article references

We analyzed all empirical studies that tested the effectiveness of HV programs for families using the touchpoints approach (with anticipatory guidance), the Neonatal Behavioral Assessment Scale (NBAS), and the Newborn Behavioral Observations (NBO). Following PRISMA guidelines [20], we conducted a comprehensive search (by title and abstract) of empirical studies conducted in any country using PsycINFO, ERIC, PsycArticles, Psychology and Behavioral Sciences Collection, MLA, Education Research Complete, Sociology Source Ultimate, and PUBMED (Supplemental File 1). We conducted Boolean searches of each database using the following search term combinations: (“neonatal behavioral observation” OR NBO OR “anticipatory guidance” OR NBAS OR “neonatal behavioral assessment scale” OR Brazelton) AND (“home treatment”, OR “home-visit*” OR “health intervention” OR “health visit*”). Forty articles were identified. We also consulted Google Scholar, which enabled us to identify another 38 records. Fifty-nine records were identified from the references. Among the 16 studies included in the review by Barlow and colleagues [17], four studies used HV. However, of these studies, we included in our review only the two that used HV as a treatment and discarded the remaining two studies because HV was only performed to evaluate treatments previously conducted in clinical or hospital settings. After the screening, 19 records were included in the review (see Fig. 1).

### Study selection

The records search was conducted by the first and third authors. To avoid double counting of the same studies, duplicate publications were identified and removed. Then, all articles were reviewed separately by the first and third authors, who checked the titles and abstracts and removed irrelevant studies according to the inclusion and exclusion criteria. The full-text screening was used when there was uncertainty about the relevance of studies after screening the title and abstract, or when they were judged to be potentially relevant (*K* = 0.80). Disagreements were resolved through joint data review and coding by consensus.

### Data extraction

Relevant data were extracted from each study, including (a) study characteristics (e.g., authors, year, and country of publication); (b) demographic characteristics of participants (e.g., number of participants, mean age of mothers and presence of risk factors); (c) methodological characteristics (e.g., recruitment method, number of HV, age of the child during HV program, intervention method, and research design); and (d) treatment effects on infants, mother, child-mother interaction, and home visitors (Brazelton programs in the various studies were extracted and considered statistically significant if the *p* value was less than 0.05). Risk factors included the presence of a depressed or adolescent mother, child developmental delay, racial/ethnic minority membership, low socioeconomic status (SES), and lack of health insurance. Intervention methods included NBO, NBAS, AG, and integrated approaches (IAs). In our review, IA referred to HV programs that integrated Brazelton’s techniques with perinatal dyadic psychotherapy in the studies by Goodman and colleagues [21, 22] and the Steps Toward Effective Enjoyable Parenting program in the study by Guthrie and colleagues [23]. Overall, all research designs were classified into randomized controlled trials, quasi-experimental studies, and feasibility studies. The first author used a form developed by the research team to extract the data. The third author verified that all extracted data were accurate and complete. Disagreements were resolved through discussion.

### Quality assessment

The studies underwent a quality assessment using Jadad’s 3-point scale [24]. The Jadad scale assesses whether studies are described as randomized (1 point), double-blinded (1 point), and whether they provide a description of withdrawals and dropouts (1 point), for a maximum of 3 points. However, we considered the blinding score 1 if the evaluator was blinded since it is usually impossible to have a double-blind design in psychological interventions. As a result, eleven studies received a Jadad score of 2 or 3, whereas eight studies received a Jadad score of 0 or 1 (see table in Supplemental File 2).

## Results

Nineteen papers (sixteen articles and three dissertations) published between 2009 and 2022 were identified (see table in Supplemental File 2). Thirteen studies were conducted in the USA, one in Australia, two in Norway, two in Denmark, one in Iceland, and one in Portugal. Regarding the presence of family risk factors, four studies involved depressed mothers, two studies involved mothers with symptoms of anxiety or depression, two studies involved children with developmental delay, and one study involved depressed mothers with children who had developmental delay. Other risk factors included being an adolescent mother in one study and having low SES, and being part of a racial/ethnic minority in four studies. Of the latter four, one included families without health insurance. Finally, four studies did not include at-risk families.

Seven studies recruited participants in a hospital after delivery, ten studies recruited participants in a clinic (e.g., prenatal clinic or family health center), three studies used a community sample, and one study did not provide information on the recruitment process. Among the studies, the number of home visits varied widely from one to thirty-three. The age of the children during the interventions ranged from less than 1 month (i.e., immediately after birth) to 24 months. Regarding the method, NBO administration was used in nine studies, NBAS in one study, AG in five studies, both AG and NBO in two studies, and finally, a combination of the three administrations of AG, NBO, and NBAS with other approaches described above in three studies.

The following sections review existing studies focusing on the main improvements in children, parents, and home visitors associated with different HV programs based on the Brazelton approach (research question 1), variation in outcomes by different categories of at-risk families (research question 2), and general characteristics of existing research studies on these programs (research question 3).

### Children outcomes

Results on the impact of the Brazelton approach on children’s adjustment are mixed (see Table 1). Available studies have focused primarily on four areas: immunization rate and the child’s motor, cognitive, and social development. *Motor development* included gross motor skills assessed with the Battelle Developmental Inventory (BDI [26]) and locomotor development assessed with the Schedule of Growing Skills II (SGS-2 [27]). *Cognitive development* included language skills such as vocabulary measured with the Communicative Development Inventory [28], perception/concepts and attention/memory measured with the BDI [26], and speech and language measured with the SGS-2 [27]. *Social development* included social-emotional, communication, cooperation, and interaction skills. Socio-emotional skills were measured with the Ages and Stages Questionnaire Social-Emotional scale [29]. The BDI Communication and Personal-Social scale [26] was used to examine communication skills. Cooperation skills were measured with the CARE-Index Cooperativeness scale [30], whereas interaction skills were measured with the SGS-2 Interactive/Social scores [27].

**Table 1:** Significant and nonsignificant improvements of children in families receiving a Brazelton home visiting treatment, by method and risk factor

**Method**	**Risk factor(s)**	**Significant improvements**	**Nonsignificant improvements**
**AG**	Low SES	**Immunization**^**a**^	
	Ethnic minority	**Cognitive development**^**b**^	
	Mixed risks and no risk	Motor development^c^	Cognitive development^d^
			Social development^e^
**NBO**	No risk	Social development^f^	Social development^g^
	Mother’s depression	**Cognitive development**^**h**^	**Motor development**^**i**^
	Child’s developmental delay		**Social development**^**j**^
	Mother’s depression Mother’s anxiety		**Cognitive development** **Motor development** **Language development** **Social development**^**k**^
**NBAS**	No risks		**Social development**^**l**^

The improvements are reported as significant at *p* < 0.05. Names in bold indicate the improvements (or lack of improvements) associated with Brazelton home visiting treatments based on randomized, single-blind studies reporting sample retention rates (Jadad score = 3)

*AG* (anticipatory guidance)*. Significant improvements*: ^a^State of vaccination [25, 37], ^b^improved vocabulary and reduced developmental delay [25, 37]; ^c^locomotor development [31]. *Nonsignificant improvements:* ^d^Development of cognition, vision, hearing, speech and language [31]. ^e^child's social response to the stimuli in the environment (interactive social skills) and self-help abilities (self-care social skills) [31]

*NBO* (Newborn Behavioral Observations)*. Significant improvements:* ^f^Cooperation [34]; ^h^perception/concept and attention/memory [32]. *Nonsignificant improvements:* ^g^Self-regulation, compliance, adaptive functioning, autonomy, affect, social communication and interaction with parents [35]; ^i^gross motor skills [32]; ^j^social development milestones (e.g., smiles, snuggles, lifts arms to be picked up) [32]; ^k^physical, cognitive, social-emotional, linguistic, and behavioral milestones as measured by the Bayley-III Scales [33]

*NBAS* (Newborn Behavioral Assessment Scale)*. Nonsignificant improvements:* ^l^Cooperation [36]

In one quasi-experimental study by Soares [31], the application of AG in HV was associated with greater motor development in children. Three experimental studies have tested the impact of NBO on social development [32–34]. Two of these [32, 33] found no differences between experimental and control samples, whereas the third [34] reported significantly higher cooperation scores in experimental samples. A large cluster randomized study using the NBO [35] and two quasi-experimental studies using the NBAS [36] and the AG [31] found no significant differences in social development between children of parents who received the Brazelton program and those who did not.

Four studies have reported cognitive improvement in children of parents who received a Brazelton intervention. Specifically, the application of AG in US samples was found to be significantly associated with higher vocabulary levels in two quasi-experimental studies [25, 37] and with higher perception/concept and attention/memory scores in an experimental study [25]. In a US experimental study, the application of NBO was associated with improvements in cognitive performance related to perception/concept and attention/memory [32]. In contrast, in a quasi-experimental study conducted in Portugal [31] and an experimental study conducted in Australia [33], no significant differences were reported in scores related to language and cognition. Finally, the application of AG was significantly associated with improvements in immunization rates in one experimental and two quasi-experimental studies conducted in the USA [25, 37].

### Mothers’ psychological outcomes

The available studies have mainly focused on three types of psychological outcomes of mothers: *satisfaction* with the intervention, *confidence* (e.g., resilience, self-esteem, and security), and *psychopathology* (e.g., depression and anxiety) (see Table 2). Of three studies [31, 38, 39], only one reported that mothers who received the Brazelton approach reported higher levels of *satisfaction* than those in control groups [31]. In these studies, satisfaction is not a part of the intervention process, but an outcome of the interventions in which the experimental groups were compared with control groups that received another active intervention or observation by a health care provider.

**Table 2:** Significant and nonsignificant improvements in mothers receiving a Brazelton home visiting treatment, by method and risk factor

**Method**	**Risk factors**	**Significant improvements**	**Nonsignificant improvements**
**AG**	Low SES Ethnic minority	**Confidence**^**a**^	
	Mixed risks and no risk	Satisfaction^b^	
**NBO**	No risk		Confidence^c^ Depression Satisfaction^d^ Stress
	Mother’s depression	Depression	
	Mother’s depression Child’s developmental delay	**Depression**	
	Mother’s depression Mother’s anxiety	**Anxiety**	**Depression**
**NBAS**	No risk		**Confidence**^**e**^
**NBO** **AG**	Child’s developmental delay		Satisfaction^f^
**NBO** **AG** **IA**	Mother’s depression	Depression Anxiety Confidence^g^	**Depression** **Anxiety** **Confidence**^**h**^ **Stress**

*AG* anticipatory guidance, *NBO* Newborn Behavioral Observations, *NBAS* Newborn Behavioral Assessment Scale, *IA* integrated approach. The improvements are reported as significant at *p* < 0.05. Names in bold indicate the improvements (or lack of improvements) associated with Brazelton home visiting treatments based on randomized, single-blind studies reporting sample retention rates (Jadad score = 3)

*AG. Significant improvements: *^*a*^Perceived resilience and self-esteem [25, 37]; ^b^trust/caring, relational/emotional, collaboration/partnership [31]

*NBO. Significant improvements*: Depression [32, 41]; anxiety [33]. *Nonsignificant improvements:* ^c^Perceived maternal efficacy [35]; depression [33, 35, 39]; ^d^satisfaction on learning child’s signals and needs in everyday situations [39]; stress [39]

*NBAS. Nonsignificant improvements:* ^e^Maternal representation [36]

*NBO–AG. Nonsignificant improvements:* ^f^Perceived quality of support intervention [38]

*NBO–AG–IA. Significant improvements:* Depression [22]; anxiety [22]; ^g^maternal self-esteem [22]. *Nonsignificant improvements:* Depression [21]; anxiety [21]; ^h^maternal self-esteem [21]; stress [21]

Regarding *confidence*, four studies [22, 25, 37] showed that mothers receiving the Brazelton approach had higher perceived resilience and self-esteem than those in the control groups. In contrast, three studies [21, 33, 36] found no significant differences in self-esteem and self-confidence. Finally, regarding *psychopathology*, a distinction must be made between studies using clinical and community samples. Some studies have used the Brazelton approach with mothers at risk for postpartum depression. Some of these studies used an NBO intervention [32, 33, 40, 41], while others used an integrated approach including AG and NBO [21, 22]. Overall, two randomized control trials [32, 41] and one quasi-experimental study [22] showed that mothers at risk for postpartum depression had reduced depressive symptoms after a Brazelton intervention. However, two experimental studies did not find the same significant differences [21, 33]. Of these two, while one study showed no differences between experimental and control groups in anxiety symptoms among depressed mothers [21], the other one that included mothers with depression or anxiety symptoms showed that receiving an NBO program significantly reduced mothers’ anxiety levels but not depression [33]. Among studies that did not use clinical samples, one experimental study [35] and one quasi-experimental study [39] found no differences between mothers in the experimental and control groups on any outcome (i.e., depressive symptoms, satisfaction, stress, and self-confidence).

### Mother-infant interaction outcomes

Studies examining the mother-infant relationship have consistently shown a positive impact of HV Brazelton programs on several outcomes, some of which we have categorized into *knowledge*, *sensitivity,* and *resources*. Outcomes related to *knowledge* include mothers’ knowledge, interest, and awareness of resources for their babies and early childhood care skills. *Sensitivity* characterizes mothers’ sensitivity to their relationship with their children in terms of reciprocity, availability, attachment quality, responsiveness, and emotional involvement. Finally, *resources* indicate the identification of environmental resources that can foster children’s development (see Table 3).

**Table 3:** Significant and nonsignificant improvements in mother-infant interaction in families receiving a Brazelton home visiting treatment, by method and risk factor

**Method**	**Risk factors**	**Significant improvements**	**Nonsignificant improvements**
**AG**	Low SES Ethnic minority	**Knowledge**^**a**^ **Resources**^**b**^	
	Mixed risk and no risk	Sensitivity^c^ Interaction^d^	
**NBO**	No risk	Knowledge^e^	Reflective function^f^ Sensitivity^g^ Interaction^h^
	Mother’s depression Mother’s anxiety	**Knowledge**^**i**^	
**NBAS**	No risk		**Sensitivity**^**j**^
**NBO** **AG**	Child’s developmental delay	Satisfaction^k^	
**NBO** **AG** **IA**	Mother’s depression		**Sensitivity**^**l**^
**AG** **IA**	Low SES Ethnic minority No Insurance	Resources^m^ Sensitivity^n^	Acceptance^o^ Organization^p^ Involvement^q^ Variety of stimulation^r^

*AG* anticipatory guidance, *NBO* Newborn Behavioral Observations, *NBAS* Newborn Behavioral Assessment Scale, *IA* integrated approach. The improvements are reported as significant at *p* < 0.05. Names in bold indicate the improvements (or lack of improvements) associated with Brazelton home visiting treatments based on randomized, single-blind studies reporting sample retention rates (Jadad score = 3)

*AG. Significant improvements: *^*a*^Parenting knowledge of nurturing practices and childrearing beliefs (i.e., empathy, developmental expectations, and use of noncorporal discipline) [25, 37], ^b^adequacy of family needs and resources (i.e., basic needs, parenting needs, interpersonal needs) [25, 37]; ^c^reciprocity, emotional availability, and attachment quality [31]; ^d^easier interaction [31]

*NBO. Significant improvements:* ^e^Concerning the infant’s communication skills, how to respond to cues, and how to establish a relationship, soothe the infant, and regulate the infant’s sleep [35, 39]; ^i^about infant’s communication, visual attention, and mutual gaze, tiredness, regulation, and verbal and non-verbal expression [33]. *Nonsignificant improvements:* ^f^Prementalizing modes, certainty about mental states, interest, and curiosity in mental states [39]*;* ^g^mother’s pleasure in interacting with her baby, the mother’s level of irritation towards the baby, and the quality of the maternal bonding [39], satisfactory maternal sensitivity versus unresponsive or controlling behavior [34]; ^h^interaction quality [35]

NBAS. *Nonsignificant improvements:* ^j^Perception of the new baby [36]

*NBO–AG Significant improvements*: ^k^For parent-infant social interaction [38]

*NBO–AG–IA. Nonsignificant improvements:* ^l^Reciprocity mother-infant [21]

AG–IA. *Significant improvements:* ^m^Appropriate learning materials for physical development, eye-hand coordination, and cognitive development [23]; ^n^responsivity in praising the child, showing affection, and reacting positively to the child’s vocal expression [23]. *Nonsignificant improvements:* ^o^Acceptance of the child [23], ^p^organization of the physical and temporal environment [23], ^q^parental involvement with the child [23], ^r^variety in daily stimulation [23]

Regarding the findings related to *knowledge*, most studies reported that mothers who received a Brazelton intervention increased their knowledge about their children compared to mothers in control groups [25, 33, 35, 37, 39, 40]. However, in only one study was the receipt of the Brazelton program significantly associated with a decrease in mothers’ curiosity and interest in interpreting children’s mental states [42].

Seven studies measured *sensitivity* outcomes and presented mixed results. Specifically, two studies reported that Brazelton interventions were associated with increased responsiveness [23] and sensitivity [31]. However, five studies [21, 34, 36, 39, 40] found no significant improvements in mother-infant relationships, sensitivity/reciprocity, emotional availability, and attachment quality.

Finally, very few studies have analyzed *resource*-related outcomes. Receiving a Brazelton intervention helped parents meet family needs, obtain resources [25, 37], and identify appropriate learning materials [23].

### Home visitor outcomes

We identified four studies that evaluated the impact of the Brazelton approach on home visitors (see Table 4). In one study [43], home visitors who used AG were more satisfied with supervision and more confident in their practice. Two experimental studies showed mixed results. McManus and Nugent [44] showed that home visitors, after using the NBO approach on infants with developmental delays, reported increased confidence but not increased knowledge about infants, as assessed by the Index of Practitioner Knowledge and Skills Scale. In contrast, Kristensen et al. [45] showed that home visitors using the NBO approach had significantly higher knowledge of infant self-regulation and intention, but not higher self-efficacy in working with early parent-infant relationships.

**Table 4:** Significant and nonsignificant improvements in home visitors delivering a Brazelton home visiting treatment, by method and risk factor

**Method**	**Risk factors**	**Significant improvements**	**Nonsignificant improvements**
**AG**	Teen mom	Confidence^a^	Job satisfaction
**NBO**	No risk	Knowledge^b^	Intention^c^ Confidence^c^ Observation skills^d^
**NBO** **AG**	Child’s developmental delay	Confidence^e^	Knowledge^f^

*AG* anticipatory guidance, *NBO* Newborn Behavioral Observations. The improvements are reported as significant at *p* < 0.05. The findings are not based on randomized, single-blind studies reporting sample retention rates (Jadad score < 3)

*AG. Significant improvements*: ^a^Related to touchpoints and self-evaluation ability [43]. *Nonsignificant improvements:* Job satisfaction [43]

*NBO. Significant improvements*: ^b^Knowledge of infant self-regulation [45]. *Nonsignificant improvements:* ^c^Referred to working with early parent-infant relationships [45]; ^d^observation skills to assess the quality of mother-infant relationships [45]

*NBO–AG. Significant improvements*: ^e^Confidence in their abilities [44]. *Nonsignificant improvements:* ^f^Knowledge about infants’ state [44]

## Discussion

This scoping review summarizes the results of 19 studies on the impact of Brazelton home visiting (HV) intervention on child development, mothers’ psychological well-being, mother-infant relationships, and home visitors’ satisfaction.

Overall, there is no clear picture regarding the impact of the Brazelton approach on children’s development. Studies with the highest Jadad scores (i.e., randomized, single-blind studies reporting sample retention rates) have suggested that AG has a positive impact on the cognitive development and immunization level of children in low SES and ethnic minority families [25]. In families who received the NBO approach, no improvement was found in children with depressed and anxious mothers [33]. However, improvement in cognitive development was found when children had a developmental delay in addition to depressed mothers [32].

Results on the impact of the Brazelton program on maternal psychological variables are also mixed. It is worth noting that, according to the studies with the highest Jadad scores, the Brazelton approach was associated with a reduction in depressive symptoms in samples of mothers at risk for postpartum depression who had children with developmental delay [32], but not in families with no risk factors [35, 39] or where the only risk factor was maternal depression [21, 33]. These findings are partially consistent with the meta-analytic results of Barlow and colleagues [17] who found no evidence of the impact of the Brazelton program on maternal depression. One might speculate that the impact of the Brazelton approach on maternal depression would be more detectable using a sample of families where both mothers and children are vulnerable.

Consistent evidence was reported in all the studies that the Brazelton approach increases parents’ knowledge about their children [25, 33, 35, 37, 39]. Indeed, in Brazelton’s perspective, both NBO and AG have the main purpose of educating parents about the peculiarities of their children’s developmental stages [11]. Interestingly, Erlingsdóttir’s study [42] reported a significant decrease, among mothers who received the Brazelton program, in curiosity and interest in interpreting their children’s mental states. However, interest and curiosity were measured using items such as *I wonder a lot about what my child is thinking and feeling*. It could be speculated that this item measures, rather than interests, parents’ doubts about their children’s behaviors. These doubts generally decrease as parents’ knowledge about their children improves. Therefore, it is likely that the Brazelton program, by promoting parents’ knowledge, may decrease their doubts about their children [46].

Contrary to expectations that increased knowledge of infants would be associated with increased parental sensitivity and confidence in their caregiving skills, no clear impact of the Brazelton approach on the sensitivity and emotionality of the mother-infant relationship could be identified. This is consistent with the mixed findings described above about the associations between the Brazelton intervention and the children’s socio-emotional development. Future studies could investigate whether maternal sensitivity and responsiveness could involve aspects of the relationship that would be less likely to be modified than mere knowledge of the infant.

Finally, available studies have reported that the Brazelton approach was significantly associated with the identification of family resources that can help children’s development (e.g., non-profit local organizations that can provide play and learning materials or offer social services). However, only three studies have examined this association [23, 25, 37] and only one was randomized and single-blind [25]. Future studies on this line of research are therefore recommended.

Methodologically, more consensus is needed regarding how to measure the impact of the Brazelton HV approach. Inconsistency in findings among studies can be attributed to common method biases as already suggested by Barlow and colleagues in their review and meta-analysis [17]. For example, the use of different measures assessing similar constructs and the varying number of home visits across studies (from one to thirty-three) would generate different and incomparable results. In addition, given that multiple home visits may be costly, identifying the optimal number of home visits to produce improvements in parenting and child development in different at-risk and non-at-risk targets could have considerable economic implications on how best to leverage this type of intervention. For the same reasons, the association between the duration of home visits and the effectiveness of the intervention needs to be clarified.

Although existing research makes a valuable contribution to understanding the impact of the Brazelton approach, other methodological inconsistencies among studies lead to caution in interpreting the results. For example, there is variability in the presence of participant risk characteristics among studies, some studies did not use an experimental research design, and others were limited by small sample sizes. Therefore, to strengthen confidence in the benefits associated with the Brazelton program, more studies with a consistent methodological approach to the selection of participant risk characteristics, research design, duration and number of home visits, and measures are needed. Only six studies presented in this paper were described as randomized and single-blinded and reported a description of withdrawals and dropouts. More studies that limit factors such as placebo effects or selection bias are needed to shed further light on the reported mixed results regarding the effectiveness of the Brazelton approach.

Another major area where further research is needed is a more thorough examination of the impact of the Brazelton approach in combination with other approaches. For example, among the studies reviewed, the use of AG in combination with other approaches was associated with lower depression and anxiety, higher self-esteem [22], greater knowledge about the appropriate learning materials for the children, and greater parental responsiveness [23]. These results highlight the need to create a more precise and standardized HV program based on the Brazelton approach, specifying the use, extent, and timing of NBO, AG, and NBAS interventions and specific integrations of other approaches. Therefore, the inclusion of studies based on integrated approaches is a limitation of the review, as studies based on integrated approaches do not allow us to distinguish the relevance of the specific Brazelton program.

The research findings collected help underscore the importance of research on preventive interventions such as the Brazelton HV programs. Specifically, this review aims to provide researchers and practitioners with valuable information to consider in their research, prevention, and intervention activities aimed at improving family well-being. Although HV programs may have an excessive cost, in the long run, these programs would be cost-effective for public spending: They could prevent future psychopathological sequelae and enable parents and their children to achieve greater well-being, resulting in reduced access to social services and healthcare [47].

## Supplementary Information

Below is the link to the electronic supplementary material.

Supplementary file1 (DOCX 16 KB)

Supplementary file2 (DOCX 53 KB)

## Abbreviations

BDI: Battelle Developmental Inventory
SGS: Schedule of Growing Skills
HV: Home visiting
IA: Integrated approaches
NBAS: Newborn Behavioral Assessment Scale
NBO: Newborn Behavioral Observations system
PRISMA: Preferred Reporting Items for Systematic Reviews and Meta-analyses
SES: Socioeconomic status
