# Supplementary material for: Home-visiting programs based on the Brazelton approach: a scoping review
Source: Eur J Pediatr. 2023 Jun 7;182(8):3469–79. doi: 10.1007/s00431-023-05048-3 (PMC10460298; doi:10.1007/s00431-023-05048-3)
Supplement: Supplementary file 1 — Supplementary file1 (DOCX 16 KB) [file 431_2023_5048_MOESM1_ESM.docx]

Supplemental File 1

Search Strategy

| **Database** | **Strategy** |
| --- | --- |
| **Psyc-INFO**  **ERIC**  **PsycArticles**  **Psychology, and Behavioral Sciences Collection**  **MLA**  **Education Research Complete,**  **Sociology source ultimate**  **(EBSCO)** | AB ( “neonatal behavioral observation” OR NBO OR "anticipatory guidance" OR NBAS OR “neonatal behavioral assessment scale” OR Brazelton ) +  AND  AB ( “home treatment” OR "home-visit*" OR "health intervention" OR "health visit*" ) |
| **PUBMED** | ((“neonatal behavioral observation” [Title/Abstract] OR "NBO"[Title/Abstract] OR "anticipatory guidance"[Title/Abstract] OR "NBAS"[Title/Abstract] OR "neonatal behavioral assessment scale"[Title/Abstract] OR "Brazelton"[Title/Abstract])  AND  ("home treatment"[Title/Abstract] OR "home visit*"[Title/Abstract] OR "health intervention"[Title/Abstract] OR "health visit*"[Title/Abstract])) AND ((clinicaltrial[Filter] OR randomizedcontrolledtrial[Filter])  AND  (english[Filter] OR french[Filter] OR italian[Filter] OR spanish[Filter])) |
| **Google Scholar** | allintitle: “neonatal behavioral observation” OR "NBO" OR "anticipatory guidance" OR "NBAS" OR “neonatal behavioral assessment scale” OR "Brazelton" |
