# Supplementary material for: Home-visiting programs based on the Brazelton approach: a scoping review
Source: Eur J Pediatr. 2023 Jun 7;182(8):3469–79. doi: 10.1007/s00431-023-05048-3 (PMC10460298; doi:10.1007/s00431-023-05048-3)
Supplement: Supplementary file 2 — Supplementary file2 (DOCX 53 KB) [file 431_2023_5048_MOESM2_ESM.docx]

**Supplemental File 2**

*Table of studies with sample descriptions, home vising intervention, outcome measures, and treatment effect*

| Study | | | Participants | | | HV intervention | | | | | Outcome Measures | | | Outcomes:  Significant improvements at p < 0.05  (Non-significant improvements at p > 0.05) | | | | Jadad scale |
| --- | --- | --- | --- | --- | --- | --- | --- | --- | --- | --- | --- | --- | --- | --- | --- | --- | --- | --- |
| Authors | Year | Country | N | M  Average age | Risk | Recruitment | N. HV | Range child’s age during intervention *months* | Method | Research design | Infants | Parents | Visitors | Inf | M variables | M- Inf interaction | HVisitor |  |
| Erlingsdóttir | 2019 | Iceland | 29 I; 26 C | 27.6 | M Depression & Anxiety | Clinic | 3 | 0-1 | NBO | RCT |  | EPDS; PAI; PRFQ |  |  |  | Lower Interest & Curiosity on Infants* |  | 2 |
| Zajicek-Farber | 2009 | USA | 35 I; 30 C | 22,5 | Low SES; Ethnic minority | Clinic | 33; 112 Phone Contacts | 0-18 | AG | QE | EPSDT;  ASQ;  CDI; | FRS; AAPI-2; RAS |  | Immunization*; Cognitive* | Confidence* | Knowledge*; Resources* |  | 1 |
| Zajicek-Farber | 2010 | USA | 35 I; 30 Static C  38 I; 35 C | 22,5 | Low SES; Ethnic Minority | Clinic | 33 | 0-18 | AG | QE + RCT | EPSDT;  ASQ;  CDI | FRS; AAPI-2; RAS |  | Immunization*; Cognitive* | Confidence* | Knowledge*; Resources* |  | 1-3 |
| Forstadt | 2012 | USA | 112 H Visitors | <18 | Teen M | *Multiple* | NR | Prenatal-3 | AG | QE |  |  | JGS; AJDI;  PSAS; OQ; |  |  |  | Self-evaluation Ability*;  (Job Satisfaction) | 1 |
| Goodman  et al. | 2013 | USA | 6 I | 32 | M Depression | Hospital | 8 | 1-3 | AG, NBO & IA | QE |  | EPDS; SCID; STAI; MSRI |  |  | Depression*; Anxiety*;  Confidence* |  |  | 2 |
| Goodman et al. | 2015 | USA | 21 I;  21C | 30.69 | M Depression | Hospital | 8 | 1-3 | AG, NBO & IA | RCT |  | EPDS; SCID; STAI; MSRI; PSI-SF; CIB |  |  | (Depression; Anxiety  Confidence; Stress) | (Sensitivity) |  | 3 |
| Greve  et al. | 2018 | Norway | 14 IG | NR | M  Depression | Clinic | 3 | 0 -1 | NBO | Feasibility |  | EPDS; PAI; RSS; NBO-PSF; EA |  |  | (Depression) | Knowledge*  (Sensitivity) |  | 1 |
| Guthrie  et al. | 2009 | USA | 33 I; 39 C | 25 | Etnic Minority Low SES; No HI | Clinic | 6 | 0-3 | AG & IA | QE | - | AAPI; IT-HOME |  |  |  | Resources*; Sensitivity*; (Acceptance; Organization; Involvement; Variety) |  | 0 |
| Høifødt  et al. | 2020 | Norway | 82 I; 114 C | 31 | No | Clinic | 1 | 0 -1 | NBO | QE | PSI-CD | EPDS; BDI; PSI-PD; MPAS; PRFQ; MCQ |  |  | (Satisfaction; Depression; Stress) | Knowledge*  (Reflective function, Sensitivity) |  | 0 |
| Killough | 2004 | USA | 19 I; 17 C | 28, 5 | No | Hospital | 2 | 0- 4 | NBAS | RCT | CARE-INDEX | MRQ; CARE-INDEX |  | (Social) | (Confidence) | (Sensitivity) |  | 3 |
| Kristensen et al. | 2019 | Denmark | 111 HVisitor | NR | No | Community | NR | NR | NBO | RCT |  |  | ISQ |  |  |  | Knowledge *  (Intention; Self-Efficacy; Observation skills) | 1 |
| Kristensen  et al. | 2020 | Denmark | 1332 I; 1234 C | 30.4 | No | Community | 12 | 0-3 | NBO | RCT | ASQ | KPCS; MDI; MABISC; Knowledge Items |  | (Social) | (Confidence; Depression) | Knowledge*  (interaction) |  | 2 |
| Mc Manus  & Nugent | 2014 | USA | 25 I; 13 C | No | DD | Clinic | 7 | 1-3 | NBO & AG | RCT |  | HVI |  |  | (Satisfaction) | Satisfaction* |  | 1 |
| McManus & Nugent | 2011 | USA | 9 HVisitors I, 9 HVisitors C | NR | DD | Community | 8 | 2-3 | NBO & AG | Feasibility |  |  | IPKS |  |  |  | Confidence*; (Knowledge) | 2 |
| Nicolson et al. | 2022 | Australia | 40 I; 34 C | 31 | M Depression & Anxiety; | Hospital | 2 or 3 | 0-2 | NBO | RCT | BSID-III | NBO-PSF; EA; NDKQ; ANRQ; EPDS; HSP Scale; PASS; SCID-5; |  | (Cognitive; Motor; Language; Social) | Anxiety*; (Depression) | Knowledge* |  | 3 |
| Mc Manus  et al. | 2020 | USA | 16 I; 22 C | >18 | M Depression DD | Clinic | 12 | 3-6 | NBO | RCT | BSID-II;  BDI-2 | CES-D |  | Cognitive*  (Motor; Social) | Depression* |  |  | 3 |
| Nugent et al. | 2014 | USA | 51 I; 55 C | 28 | M Depression | Hospital | 1 | 0-1 | NBO | RCT |  | EPDS |  |  | Depression* |  |  | 2 |
| Nugent  et al. | 2017 | Usa | 18 I; 17 C | 21.5 | No | Hospital | 2 | 0 - 4 | NBO | RCT | CARE-INDEX | CARE-INDEX |  | Social* |  | (Sensitivity) |  | 3 |
| Soares | 2016 | Portugal | 42 I; 43 C | 30.6 | Mixed risk & no risk | Clinic | 4 | 11-24 | AG | QE | CARE –INDEX  SGS | PCRS; CARE -INDEX; |  | Motor*  (Cognitive; Social) | Satisfaction* | Interaction*;  Sensitivity* |  | 2 |

Participants: I =Intervention group, C= control M= mothers; F= fathers; HV= Home-visit; HVisitor= Home-visitor; Inf= infants; NR= not reported.

Recruitment: HC= Health Center, Antenatal Clinic.

Risk: HI= Health Insurance; DD= Developmental Delay; NR= not reported.

Method: AG= Anticipatory guidance; NBO=Newborn Behavioral Observations; IA=Integrated Approach.

Research design: RCT= Randomized Controlled Trial; QE= Quasi-Experimental.

Measures: *Children*. EPSDT= Early and Periodic Screening, Diagnostic and Treatment; ASQ= Ages & Stage Questionnaire; CDI= Communicative Development Inventories; BSID-II= Bayley Scales Infant Development-II; BDI-II= Batelle Developmental Inventory 2nd; PSI-CD= Parenting Stress Index-Child domain; SGS-II=Schedule of Growing Skills II. *Parents.* AAPI-2= Adult-Adolescent Parenting Inventory-2; BDI=Beck Depression Inventory; CES-D= Center for Epidemiology Studies Depression Scale; CIB= Coding Interactive Behavioral manual; EA= Emotional Availability; EPDS= Edinburgh Postnatal Depression Scale; FRS=Family Resources Scale; HRI-C= Helping Relationship Inventory- Client; HVI= Home-visiting Index; PCRS = Parent-Caregiver Relationship Scale; IT-HOME= Infant-Toddler HOME Inventory; KPCS= Karitane Parenting Confidence Scale; MABISC= Mother & Baby Interaction Scale; MCQ= Maternal Confidence Questionnaire; MDI = Major Depression Inventory; MPAS= Mother Postnatal Attachment Scale; MRQ= Maternal Representations Questionnaire; MSPSS= Multidimensional Scale of Perceived Social Support; MSRI= Maternal Self-Report Inventory; NBO-PSF=NBO Parent Satisfaction Form; PAI= Prenatal Attachment Inventory; PRFQ= Parental Reflective Functioning Questionnaire; PSI-PD= Parenting Stress Index Parent Domain; PSI-SF= Parenting Stress Index Short Form; RAS= Resilience Attitude Scale; RSS= Relationship Satisfaction Scale; SCID= Structured Clinical Interview; STAI= State Trait Anxiety Inventor. *Visitors*. HRI-W= Helping Relationship Inventory- Worker; JGS= Jobs General Scale; aJDI= Abridged Job Descriptive Index; IPKS= Index of Practitioner Knowledge and Skills; ISQ= Intention Self-Efficacy Questionnaire; OQ=Open Questions; PSAS= Provider Self-Assessment Scale.

Outcomes: *= statistically significant. Non-significant differences are reported in parentheses.
